# Scattered seeding of CAR T cells in solid tumors augments anticancer efficacy

**DOI:** 10.1093/nsr/nwab172

**Published:** 2021-09-21

**Authors:** Hongjun Li, Zejun Wang, Edikan A Ogunnaike, Qing Wu, Guojun Chen, Quanyin Hu, Tianyuan Ci, Zhaowei Chen, Jinqiang Wang, Di Wen, Hongwei Du, Jie Jiang, Jie Sun, Xingcai Zhang, Gianpietro Dotti, Zhen Gu

**Affiliations:** College of Pharmaceutical Sciences, Zhejiang University, Hangzhou 310058, China; Liangzhu Laboratory, Zhejiang University Medical Center, Hangzhou 310058, China; Department of Bioengineering, University of California, Los Angeles, CA 90095, USA; Jonsson Comprehensive Cancer Center, University of California, Los Angeles, CA 90095, USA; California NanoSystems Institute, University of California, Los Angeles, CA 90095, USA; Department of Bioengineering, University of California, Los Angeles, CA 90095, USA; Jonsson Comprehensive Cancer Center, University of California, Los Angeles, CA 90095, USA; California NanoSystems Institute, University of California, Los Angeles, CA 90095, USA; Center for Nanotechnology in Drug Delivery, Eshelman School of Pharmacy, University of North Carolina, Chapel Hill, NC 27599, USA; Lineberger Comprehensive Cancer Center, University of North Carolina, Chapel Hill, NC 27599, USA; College of Pharmaceutical Sciences, Zhejiang University, Hangzhou 310058, China; Department of Bioengineering, University of California, Los Angeles, CA 90095, USA; Jonsson Comprehensive Cancer Center, University of California, Los Angeles, CA 90095, USA; California NanoSystems Institute, University of California, Los Angeles, CA 90095, USA; Joint Department of Biomedical Engineering, University of North Carolina at Chapel Hill and North Carolina State University, Raleigh, NC 27695, USA; Department of Bioengineering, University of California, Los Angeles, CA 90095, USA; Jonsson Comprehensive Cancer Center, University of California, Los Angeles, CA 90095, USA; California NanoSystems Institute, University of California, Los Angeles, CA 90095, USA; College of Pharmaceutical Sciences, Zhejiang University, Hangzhou 310058, China; Department of Bioengineering, University of California, Los Angeles, CA 90095, USA; Jonsson Comprehensive Cancer Center, University of California, Los Angeles, CA 90095, USA; California NanoSystems Institute, University of California, Los Angeles, CA 90095, USA; College of Pharmaceutical Sciences, Zhejiang University, Hangzhou 310058, China; Department of Bioengineering, University of California, Los Angeles, CA 90095, USA; Jonsson Comprehensive Cancer Center, University of California, Los Angeles, CA 90095, USA; California NanoSystems Institute, University of California, Los Angeles, CA 90095, USA; Department of Bioengineering, University of California, Los Angeles, CA 90095, USA; Jonsson Comprehensive Cancer Center, University of California, Los Angeles, CA 90095, USA; California NanoSystems Institute, University of California, Los Angeles, CA 90095, USA; Lineberger Comprehensive Cancer Center, University of North Carolina, Chapel Hill, NC 27599, USA; Department of Microbiology and Immunology, University of North Carolina, Chapel Hill, NC 27599USA; Department of Cell Biology and Bone Marrow Transplantation Center of the First Affiliated Hospital, Zhejiang University School of Medicine, Hangzhou 310058, China; Department of Cell Biology and Bone Marrow Transplantation Center of the First Affiliated Hospital, Zhejiang University School of Medicine, Hangzhou 310058, China; John A. Paulson School of Engineering and Applied Sciences, Harvard University, Cambridge, MA 02138, USA; Lineberger Comprehensive Cancer Center, University of North Carolina, Chapel Hill, NC 27599, USA; Department of Microbiology and Immunology, University of North Carolina, Chapel Hill, NC 27599USA; College of Pharmaceutical Sciences, Zhejiang University, Hangzhou 310058, China; Liangzhu Laboratory, Zhejiang University Medical Center, Hangzhou 310058, China; Department of Bioengineering, University of California, Los Angeles, CA 90095, USA; Jonsson Comprehensive Cancer Center, University of California, Los Angeles, CA 90095, USA; California NanoSystems Institute, University of California, Los Angeles, CA 90095, USA; Department of General Surgery, Sir Run Run Shaw Hospital, School of Medicine, Zhejiang University, Hangzhou 310016, China

**Keywords:** drug delivery, CAR T therapy, microneedle patch, solid tumor treatment, cell delivery

## Abstract

Chimeric antigen receptor T cell (CAR T) therapy was a milestone in the treatment of relapsed and refractory B cell malignancies. However, beneficial effects of CAR T cells have not been obtained in solid tumors yet. Herein, we implement a porous microneedle patch that accommodates CAR T cells and allows *in situ* penetration-mediated seeding of CAR T cells when implanted in the tumor bed or in the post-surgical resection cavity. CAR T cells loaded in the pores of the microneedle tips were readily escorted to the tumor in an evenly scattered manner without losing their activity. Such microneedle-mediated local delivery enhanced infiltration and immunostimulation of CAR T cells as compared to direct intratumoral injection. This tailorable patch offers a transformative platform for scattered seeding of living cells for treating a variety of tumors.

## Teaser

A porous microneedle patch was elaborated to load CAR T cells, and thus readily escorted CAR T cells to the solid tumor to break the physical barrier in an evenly scattered manner without losing the cells’ activity.

## INTRODUCTION

Chimeric antigen receptor (CAR) expressing T cells engineered with specific tumor-associated antigen (TAA) targeting ability have shown remarkable potency in B cell malignancies [1–4]. The US Food and Drug Administration (FDA) approved the use of CD19-targeting CAR T cells for treating pediatric acute lymphoblastic leukemia in 2017. In contrast, clinical studies with CAR T cells for solid tumors have not shown remarkable antitumor effects yet [5–8]. Solid tumors are characterized by a unique microenvironment characterized by physical and physiochemical barriers [7,9]. Abnormal vasculature, dense extracellular matrix and interstitial fluid pressure knit physical barriers preventing CAR T cells from infiltrating the tumor bed. Furthermore, immunosuppressive cells and soluble factors within the tumor microenvironment (TME) further hamper proliferation and effector function of CAR T cells [10–12].

**Figure 1.**
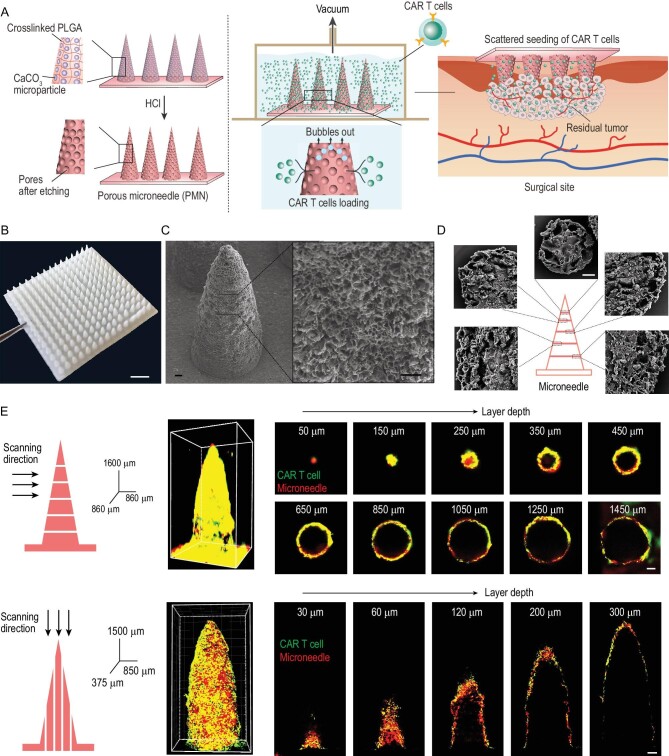
Characterization of porous microneedle (PMN) patch for CAR T cell loading and delivery. (A) Schematic of PMN fabrication, CAR T cell loading and implant of CAR-T-cell-loaded PMN (PMN@CAR T) within the tumor bed after surgery. (B) A representative picture of PMN; scale bar: 2 mm. (C) Representative SEM images of the PMN tip showing the porous structure; scale bar: 50 μm. (D) Representative SEM images of the cross section of PMN with different layer widths; scale bar: 20 μm. (E) The 3D reconstruction of the PMN@CAR T cells by confocal images. CAR T cells and microneedle patch were labeled with CFSE and rhodamine B, respectively; scale bar: 100 μm.

Surgery is a fundamental therapeutic strategy for several solid tumors. Post-surgical *in situ* administration of CAR T cells offers a potential solution for overcoming the physical barriers in solid tumors and preventing tumor recurrence [13–16]. Surgical removal of bulky tumors delays tumor recurrence, relieves physical barriers and exposes residual cancer cells to endogenous effector T cells [17]. However, the local spread of residual micro tumors after surgery poses a severe obstacle to the precise delivery of adoptively transferred CAR T cells [18–20]. Here, we describe a polymeric porous microneedle (PMN) patch that can accommodate CAR T cells and allow scattered seeding of these cells intratumorally or within the surgical tumor resection (Fig. 1A). The biocompatible poly (lactic-co-glycolic acid) (PLGA) microneedle scaffold offers sufficient mechanical force for insertion into the tumor lesion. The CaCO_3_ microparticles (diameter of ∼8 μm) in the microneedle patch are etched with

 

hydrochloric acid, leaving pores. During the insertion within the tumor, CAR T cells residing in the pores are protected from scraping. At the same time, microneedles (up to 15 × 15 arrays) provide an

 

insertion area of 144 mm^2^ with even delivery points (225 tips), ensuring ample scattered cell delivery. We hypothesized that PMN-mediated CAR T cell delivery promotes distribution and penetration of CAR T cells in solid tumors, leading to tumor eradication.

## RESULTS

### Preparation of porous microneedle patch and CAR T cell loading

The microneedle patch was fabricated by molding and polymerizing the mixture of methacryloyl chloride modified 4-arm-PLGA (Fig. S1) and triethylene glycol diacetate, along with CaCO_3_ microparticles (Fig. S2). The 15 × 15 conical microneedle has a base radius of 250 μm with 200 μm spacing and is up to 1500 μm in height (Fig. 1B). Subsequently, the patch was swelled, and CaCO_3_ microparticles were etched in hydrogen chloride dioxane solution (Fig. S3 and Movie S1 in the online supplementary file). Scanning electron microscopy (SEM) images revealed a jagged and porous surface formed after etching (Fig. 1C and Fig. S4). SEM images of the cross section of the PMN further demonstrated that the pores were formed by the etching of CaCO_3_ particles (Fig. 1D and Fig. S5). In addition, the size of the pores ranging from 5 to 20 μm is sufficient for loading CAR T cells [21]. The PMN exhibited a slightly weaker mechanical strength than the unetched microneedle, with a failure force of 2.4 N at 500 μm displacement, which can be attributed to the mechanical strength enhancement of the cross-linked structure and to the high molecular PLGA present in the formulation (Fig. S6). CAR T cells were loaded into the microneedle under vacuum (100 mbar), and bubble formation was observed during this process. No significant changes in mechanical force were detected post-loading the CAR T cells (Fig. S6).

**Figure 2.**
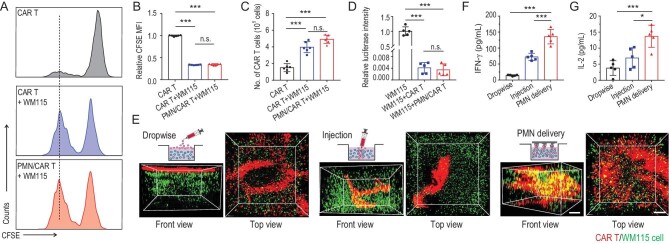
PMN promotes CAR T cell infiltration in a 3D tumor model *in vitro*. (A) CSPG4^+^ CAR T cells labeled with CFSE were co-cultured for 3 days with CSPG4-expressing WM115 tumor cells. Representative flow cytometry histograms showing CFSE dilution are presented. (B) Relative mean fluorescence intensity of CFSE in (A), indicating T cell proliferation, *n* = 6. (C) Number of CAR T cells counted by flow cytometry after incubation with WM115 cells, *n* = 6. (D) CSPG4^+^ CAR T cells were co-cultured for 3 days with firefly luciferase labeled WM115 cells. Relative luciferase intensity after 3 days of culture is illustrated, *n* = 5. (E) Schematic of three approaches (dropwise, single-needle injection and PMN) used to deliver CAR T cells into a 3D tumor model *in vitro*. The 3D reconstruction illustrates CAR T cells and WM115 cell distribution in the 3D tumor model at day 3 after T cell seeding; scale bar: 300 μm. Measurement of human (F) IFN-*γ* and (G) IL-2 released by CAR T cells in the 3D tumor model at day 3, *n* = 5. Data are presented as mean ± s.d., and statistical significance was calculated via one-way ANOVA with a Tukey post-hoc test. *P* value: ^*^P < 0.05, ^*^^*^^*^P < 0.001; n.s. means no significant difference.

We first used melanoma as a model and targeted tumor cells via CAR T cells specific for the melanoma-associated antigen chondroitin sulfate proteoglycan-4 (CSPG4) [22,23]. CAR T cells were loaded into a PMN and evaluated using a reconstructed 3D confocal image. CAR T cells (green) resided in the microneedle tip interior space through a layer-by-layer scanning of the PMN with confocal laser scanning microscope (CLSM) and 3D reconstruction (Fig. 1E). Each needle was loaded with ∼22 000 cells as calculated by flow cytometry and counting beads (Fig. S7). A loading efficiency of ∼20% was achieved when the PMN was incubated with CAR T cells at a density of 10^7^ cells/mL (Fig. S7). The biological effects of CAR T cells loaded into the PMN were compared to those of free CAR T cells by analyzing cell proliferation, cytokine release and cytotoxic activity. CAR T cells labeled with carboxyfluorescein succinimidyl ester (CFSE) were co-cultured with CSPG4 expressing human melanoma cells (WM115, Fig. S8) for 3 days [24]. CFSE dilution, indicating cell division, was observed in both the CAR T cells loaded into the PMN and free CAR T cells (Fig. 2A), and comparable CAR T cell proliferation was detected (Fig. 2B and C). Cytotoxic activity against WM115 cells (Fig. 2D), and secretion of interleukin-2 (IL-2) and interferon-*γ* (IFN-*γ*) of CAR T cells in the PMN and free CAR T cells were similarly indicating that PMNs do not damage CAR T cells (Fig. S9).

### Scattered seeding of CAR T cells promotes their proliferation and activity

We hypothesized that the array structure of the PMN could cover an extensive area and thus promote wider distribution of CAR T cells compared to single needle-mediated injection of CAR T cells. Therefore, we developed a 3D Matrigel model (cylinder, radius × height; 3.2 mm × 1.5 mm) containing tumor cells to mimic the solid tumor *in vitro* (Fig. 2E) [25]. We determined the mapping of CAR T cells with the 3D Matrigel model through dropwise, intra-gel injection, and PMN delivery. After 3 days of incubation, delivery mediated by PMN resulted in homogenous mapping of the CAR T cells, while the CAR T cells administrated by dropwise and intra-gel injection were restricted to a confined space (Fig. 2E, Movies S2–S4). Furthermore, an approximately 2-fold higher level of IFN-*γ* (Fig. 2F) and IL-2 (Fig. 2G) were secreted by CAR T cells loaded into the PMN compared to CAR T cells directly injected or deposed on the surface of the 3D-gel model, further confirming the better distribution of CAR T cells via PMN administration.

**Figure 3.**
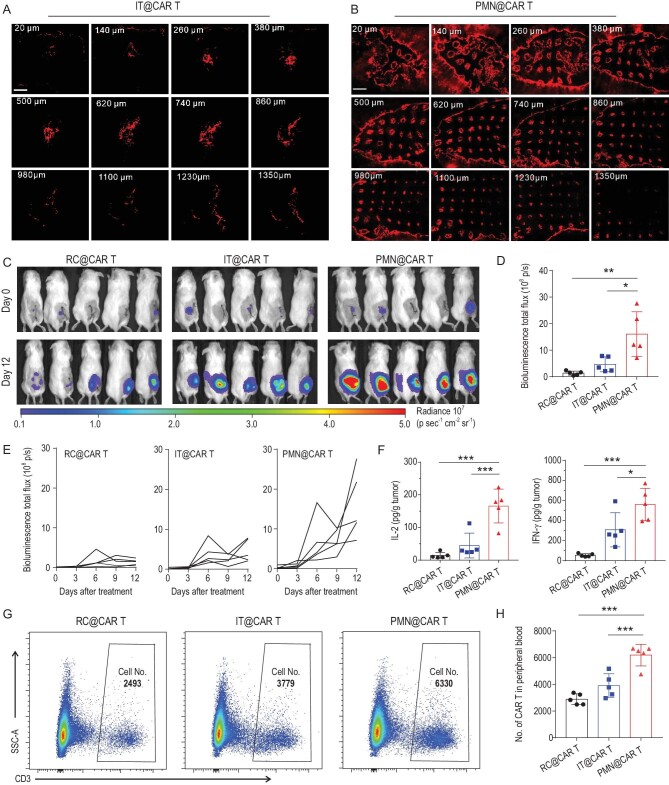
PMN promotes wider distribution of CAR T cells *in vivo*. Distribution of CAR T cells (red) within the WM115 tumor after (A) single needle intratumoral injection and (B) PMN insertion; scale bars: 1 mm. CAR T cells were pre-labeled with DiI. The number on the upper left represents the layer depth. (C) T cell bioluminescence of firefly luciferase-expressing CAR T cells inoculated within the post-surgical tumor bed. CAR T cells were delivered directly into the resection cavity, intratumorally using a single needle injection or intratumorally via PMN-assisted implant. (D) T cell bioluminescence at day 12 after treatment is illustrated, *n* = 5. (E) Kinetics of T cell bioluminescence in (C). (F) Levels of human IL-2 and IFN-*γ* detected in the tumor lysates at day 12 after CAR T cells treatment, *n* = 5. (G) Representative flow cytometric plots and (H) quantification of CAR T cells (CD45^+^CD3^+^) in the peripheral blood at day 12 after CAR T cells administration, *n* = 5. Data are presented as mean ± s.d., and statistical significance was calculated via one-way ANOVA with a Tukey post-hoc test. *P* value: ^*^P < 0.05, ^*^^*^P < 0.01, ^*^^*^^*^P < 0.001.

We then evaluated the antitumor effects of CAR T cells delivered with a PMN *in vivo*. First, we evaluated the CAR T cell release from the PMN after insertion in the tumor. More than 50% of loaded CAR T cells were delivered to the tumor within 15 min (Fig. S10). We then compared the intratumoral distribution of DiI pre-labeled CAR T cells through intratumoral injection and PMN-mediated delivery in the WM115 melanoma tumor model in NOD.Cg-Prkdc^scid^ Il2rg^tm1Wjl^/SzJ (NSG) mice. Twenty-four hours after treatment, CAR T cells were confined within the applied region when CAR T cells were inoculated intratumorally (IT@CAR T) (Fig. 3A). In contrast, CAR T cells delivered via PMN (PMN@CAR T) showed more prominent tumor infiltration (Fig. 3B). We then tested the proliferation and persistence of CAR T cells in the post-surgery model in WM115 melanoma-bearing mice. After removing 90% of the tumor, firefly luciferase-labeled CAR T cells were injected into the resection cavity (RC@CAR T), injected intratumorally as a single-needle inoculation or via PMN-assisted delivery. The proliferation of CAR T cells was monitored by *in vivo* imaging (IVIS). As shown in Fig. 3C and Fig. S11, bioluminescence signals of CAR T cells increased rapidly after implantation with PMN. Quantitative analysis of the bioluminescence showed that PMN-based delivery caused 3-fold increases of bioluminescence signals compared to other treatments (Fig. 3D and E). In parallel, PMN-based delivery of CAR T cells caused increased local expression of IL-2 and IFN-*γ* (Fig. 3F). Increased bioluminescence of CAR T cells corresponded to CAR T cell expansion because at day 12 after treatment the number of CAR T cells (CD3^+^ cells) in the PMN-treated mice was higher compared to control groups (Fig. S12). Of note, local delivery of CAR T cells via PMN caused systemic distribution of the cells because human CD45^+^CD3^+^ cells were detected in the peripheral blood (Fig. 3G and H).

### Anti-solid tumor activity of CAR T cells with scattered delivery

**Figure 4.**
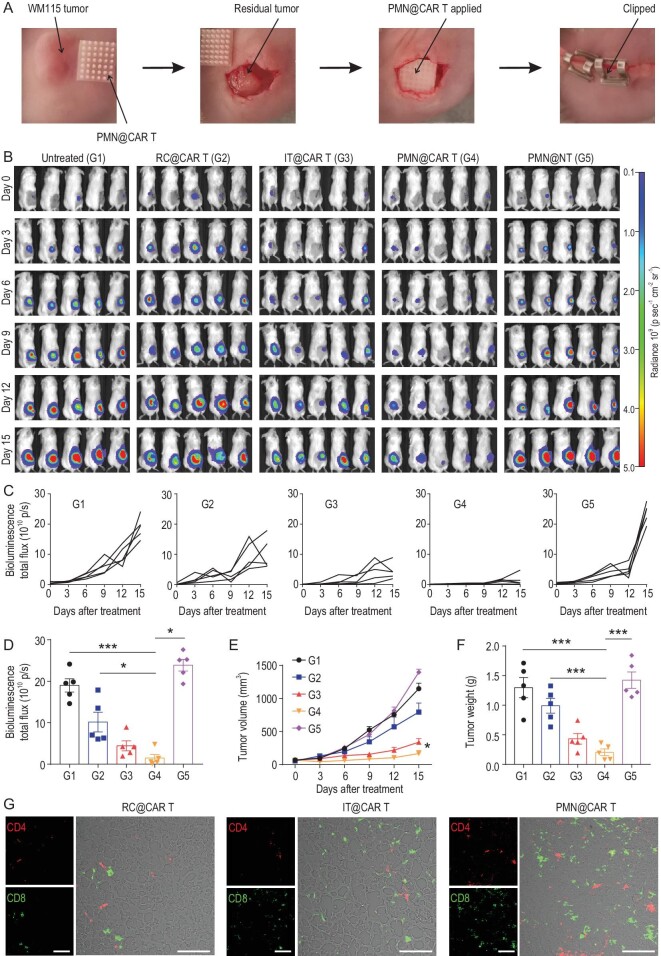
CAR T cells delivered by PMN show enhanced antitumor effects in the post-surgical resection melanoma model. (A) Schematic of the mouse model in which melanoma engrafted subcutaneously is partially resected and CAR T cells delivered within the resection cavity. (B) Representative tumor bioluminescence of WM115-bearing mice treated with CSPG4^+^ CAR T cells administered with different modalities. Untreated mice and mice treated with a PMN loaded with control T cells were used as control. (C) Kinetics of tumor bioluminescence and (D) tumor bioluminescence at day 15 post-treatment. (E) Tumor growth curve and (F) tumor weight after treatment, *n* = 5. (G) Representative immunofluorescence showing CD4^+^ and CD8^+^ T cells within the tumor after treatment; scale bars: 100 μm. Data are presented as mean ± s.d., and statistical significance was calculated via one-way ANOVA with a Tukey post-hoc test. *P* value: ^*^P < 0.05, ^*^^*^^*^P < 0.001.

We further investigated whether PMN delivery of CAR T cells could prevent tumor recurrence after partial surgery. Firefly luciferase-labeled WM115 cells were engrafted subcutaneously in NSG mice, and upon partial tumor resection CAR T cells were delivered directly into the resection cavity, intratumorally or via PMN injection. Untreated mice and mice receiving a PMN containing non-engineered T cells (PMN@NT) served as controls (Fig. 4A). Tumor bioluminescence significantly decreased in mice that received CAR T cell delivery via PMN compared with deposition and intratumoral injection (Fig. 4B–D). Tumor size (Fig. 4E) and weight (Fig. 4F) measurements also showed the superior activity of CAR T cells delivered via PMN. TUNEL assay demonstrated a higher rate of tumor cell death in mice treated with CAR T cells delivered via PMN (Fig. S13). Higher infiltration of CD8^+^ and CD4^+^ T cells was also detected using immunofluorescence staining (Fig. 4G). Taken together, these data indicate that CAR T cells delivered via PMN outperformed deposition administration and intratumoral injection methods.

**Figure 5.**
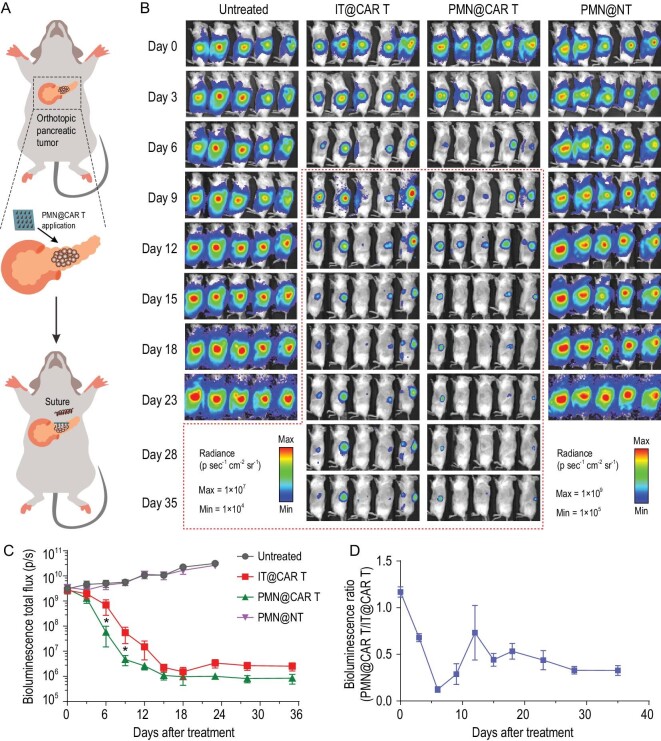
CAR T cells delivered by PMN show enhanced antitumor effects in an orthotopic pancreatic tumor model. (A) Schematic of the mouse model in which pancreatic tumor cells were engrafted in the pancreas and CAR T cells delivered intratumorally. (B) Representative tumor bioluminescence of Panc01-bearing mice treated with B7-H3^+^ CAR T cells administered with intra-tumor injection and PMN-assisted administration. Untreated mice and mice treated with a PMN loaded with control T cells were used as control. (C) Kinetics of tumor bioluminescence of IT@CAR T and PMN@CAR T after treatment, *n* = 5. (D) The ratio of bioluminescence signal of the mice that received PMN@CAR T and IT@CAR T treatment (PMN@CAR T/IT@CAR T), *n* = 5. Data are presented as mean ± s.e.m., and statistical significance was calculated via T-Test. *P* value: ^*^P < 0.05.

We further validated the applicability of the PMN delivery strategy of CAR T cells in an orthotopic pancreatic tumor model. Firefly luciferase-tagged human pancreatic cancer cells (Panc-1) engrafted in the pancreas of NSG mice, were treated with CAR T cells targeting the pancreatic-cancer-associated antigen B7-H3 [26] (Fig. 5A). B7-H3 CAR T cells were administrated by direct injection into the tumor bed or accommodated by the PMN patch after surgical exposure of the pancreatic tumor. As shown in Fig. 5B and C, the tumors in the mice treated with PMN@NT continue growing, while intra-pancreatic tumor injection and PMN delivery of CAR T cells effectively restrained the tumor growth, but PMN@CAR T cells showed superior effects, which was further confirmed by the ratio of the bioluminescence signals of mice that received treatment with PMN@CAR T/IT@CAR T (Fig. 5D).

## DISCUSSION

As a promising cancer-combating strategy, CAR T cell therapy faces several barriers that impede the clinical progress for solid tumor treatment, such as the dense physical extracellular matrix and immunosuppressive environment. In particular, physical obstacles formed by the extracellular matrix preclude sufficient infiltration of CAR T cells into the solid tumor, and inhibit the approach and recognition of CAR T cells towards tumor cells. Ongoing solutions for solid tumor treatment include employing hydrogel and implantable films to serve as the reservoirs for sustained release of CAR T cells [27–29]. Intratumor administration of CAR T cells is also under investigation in clinical trials combating solid tumors [30,31]. In comparison, PMN patch-assisted delivery enables scattered seeding of CAR T cells in the solid tumor. This microneedle patch-guided invasion of CAR T cells into the tumor matrix disrupts the physical barrier and allows the talent revelation of CAR T cells. Multipoint scattered seeding amplifies the probabilities of cell interactions between CAR T and tumor cells, and promotes CAR T cell activation and infiltration in the solid tumor matrix.

The microneedle patch has been used in the delivery of distinct drugs, including small molecules and protein drugs like galanthamine, insulin and antibodies, in a mini-invasive and transdermal manner [32–35]. To enhance therapeutic efficacy, advanced strategies such as introducing nano/micropores to the needles and integration with drug-loading CaCO_3_ microparticles have been developed [36–38]. Recently, functional cell delivery with a microneedle patch was reported for cells such as mesenchymal stem cells and dendritic cells [39,40]. These strategies extend the application of microneedles in combating various scenarios, including cancer, tissue reconstruction and medical cosmetology, by delivering live cells. Unlike small molecules and protein drugs, it is difficult for cells to penetrate certain tissues and diffuse inside them due to their large size. Transdermal delivery techniques with microneedle patches could help overcome the relative limitations of cell-based therapies. However, several challenges remain, including cell inactivation during microneedle manufacturing, insufficient cell loading, unsatisfying cell delivery efficiency and limited cell storage methods. In the present study, made from biocompatible PLGA, the PMN patch can be customizable in patch dimension and needle density depending on practical needs. Unlike the reported method that preloaded the cells before the microneedle molding, we accommodate cells in the pores of the microneedle, which could maintain their activity. The storage and large-scale manufacturing for meeting the Good Manufacturing Practice (GMP) could be more accessible than the preloading techniques. On the other hand, the PMN patch needs further optimization regarding porosity and cell loading capacity improvement, as well as balance between the porosity and mechanical force.

Overall, the microneedle patch offers a multipoint, scattered delivery tool of CAR T cell seeding that augments T cell infiltration within the tumor by overcoming poor T cell biodistribution caused by physical barriers in solid tumors. The PMN patch can be applied to the resection cavity to prevent local tumor recurrence and potential metastatic dissemination. This strategy can be extended as a local treatment platform for living cell delivery targeting a variety of diseases.

## MATERIALS AND METHODS

### Fabrication of the PMN array patch

The 4-arm-PLGA-Acry was dissolved in dioxane with a final concentration of 500 mg/mL, azodiisobutyronitrile (AIBN) was dissolved in Dioxane with a concentration of 100 mg/mL and the linear PLGA was dissolved in Dioxane with a concentration of 200 mg/mL. Then, 300 mg of 4-arm-PLGA-Acry, 150 mg of triethylene glycol diacrylate (TEGDA), 10 mg of AIBN, 20 mg of PLGA and 90 mg of CaCO_3_ microparticles were mixed and added to a polydimethylsiloxane (PDMS) micromold (Blueacre Technology Ltd.) with dioxane pre-filled into the needle. Four hours later, the microneedle was cross-linked overnight at 90°C before the microneedle patch was peeled off. The microneedle patch was placed in HCl/hexane solution for 2 hours for swelling, and then water was added to initiate the reaction between HCl and CaCO_3_, accompanied by CO_2_ bubbles generated. Finally, the PMN patch was treated with plasma to generate a hydrophilic surface. The morphology of the PMN patch was characterized by SEM (ZEISS Supra 40VP).

### *In vivo* antitumor activity

WM115 cells (5 × 10^6^) with luciferase expression were injected into the NSG mice subcutaneously. When it reached 100 mm^3^, 90% of the tumor was surgically removed. CAR T cells (1 × 10^6^) were administrated via subcutaneous injection, intratumoral injection and PMN injection to the tumor sites. After CAR T cells application, the bioluminescence signals were recorded on day 0, day 3, day 6, day 9, day 12 and day 15 with IVIS (Perkin). The analysis of signals was performed by Living Image Software. After the treatment the mice were sacrificed, and the tumor weight and images of the tumors were recorded. In the melanoma model, the microneedle patch was maintained for 15 min in the tumor before removal.

Twenty days after Panc01 cells inoculation, the progress of the orthotopic pancreatic tumor was tracked with the IVIS system by detecting the bioluminescence signals. During the microneedle-loaded CAR T cells administration, the pancreas was carefully surgically exposed by an abdomen incision. The CAR T cells-loaded PMN was carefully inserted into the tumor on the pancreas and immobilized with a biological glue (3M Vetbond Tissue Adhesive). The wound on the abdomen was carefully closed in two layers. After CAR T cells application, the bioluminescence signals were recorded on predetermined days with IVIS (Perkin).

### Statistical analysis

All results are presented as the mean ± standard deviation (s.d.) or the mean ± standard error of the mean (s.e.m.), as indicated. Tukey post-hoc tests and one-way ANOVA were used for multiple comparisons. Student T-tests were used for two group comparisons. Survival benefit was determined using a log-rank (Mantel-Cox) test. All statistical analyses were carried out with the Prism software package (PRISM 5.0; GraphPad Software, 2007). The threshold for statistical significance was *P *< 0.05.

## Supplementary Material

nwab172_Supplemental_Files
