# Supplementary material for: Scattered seeding of CAR T cells in solid tumors augments anticancer efficacy
Source: Natl Sci Rev. 2021 Sep 21;9(3):nwab172. doi: 10.1093/nsr/nwab172 (PMC8900686; doi:10.1093/nsr/nwab172)
Supplement: nwab172_Supplemental_Files [file nwab172_supplemental_files.zip › Supplementary_data.docx]

Supplementary Materials for

Scattered Seeding of CAR T Cells in Solid Tumors Augments Anticancer Efficacy

Hongjun Li^1,2,3,4,5†^, Zejun Wang^3,4,5†^, Edikan Archibong^6,7^, Qing Wu^1^, Guojun Chen^3,4,5^, Quanyin Hu^7^, Tianyuan Ci^3,4,5^, Zhaowei Chen^1,3,4,5^, Jinqiang Wang^1,3,4,5^, Di Wen^3,4,5^, Hongwei Du^6,7^, Jie Jiang^8^, Jie Sun^8^, Xingcai Zhang^9^, Gianpietro Dotti^6,7^*, Zhen Gu^1,2,3,4,5^*

**Affiliations:**

^1^ College of Pharmaceutical Sciences, Zhejiang University, Hangzhou, Zhejiang 310058, China

^2^ Liangzhu Laboratory, Zhejiang University Medical Center, Sir Run Run Shaw Hospital, Hangzhou, Zhejiang 310058, China

^3^ Department of Bioengineering, University of California, Los Angeles, California 90095, United States

^4^ Jonsson Comprehensive Cancer Center, University of California, Los Angeles, California 90095, United States

^5^ California NanoSystems Institute, University of California, Los Angeles, California 90095, United States

^6^ Joint Department of Biomedical Engineering, University of North Carolina at Chapel Hill and North Carolina State University, Raleigh, North Carolina 27695, United States

^7^ Lineberger Comprehensive Cancer Center, University of North Carolina, Chapel Hill, North Carolina 27599, United States

^8^ Department of Cell Biology and Bone Marrow Transplantation Center of the First Affiliated Hospital, Zhejiang University School of Medicine, Hangzhou, Zhejiang 310058, China

^9^ John A. Paulson School of Engineering and Applied Sciences, Harvard University, Cambridge, Massachusetts 02138, United States

*Corresponding authors: Prof. Zhen Gu, Email: guzhen@zju.edu.cn, Phone: +86-571-88208417, Fax: +86-571-88208417; Prof. Gianpietro Dotti, Email: gdotti@med.unc.edu, Phone: +1-919 9628279, Fax: +1-919 96288103

†H.L. and Z.W. contributed equally to this work.

**This PDF file includes:**

Supplementary text

Fig. S1 to S13

Legend for Movie S1-Movie S4

**Other Supplementary Materials for this manuscript include the following:**

Movie S1-S4

**Materials and Methods:**

Linear poly(lactic-co-glycolic acid) (PLGA, lactic: glycolic 75:25, WM 76,000-115,000), methacryloyl chloride, triethylamine (TEA), tin(II) 2-ethylhexanoate, hydrogen chloride solution, 4.0 M in dioxane (HCl/Hexane) were purchased from Sigma Aldrich. Lactic (LA), glycolic (GA), pentaerythritol, azobisisobutyronitrile (AIBN), triethylene glycol diacetate (TEGDA) were purchased from TCI America. Calcium carbonate microparticle (~ 6 μm) were obtained from EggTech Ltd. (Canada).

The WM115 human melanoma cells were maintained in Dulbecco’s Modified Eagle’s Medium (DMEM, Gibco) supplemented with 10% fetal bovine serum (Invitrogen), 100 U/mL penicillin (Invitrogen) and 100 U/mL streptomycins (Invitrogen). Human T cells engineered with chondroitin sulfate proteoglycan 4 chimeric antigen receptors (CSPG4 CAR T) were cultured in a complete medium containing 45% RPMI 1640 medium (Gibco) and 45% Click’s medium (Irvine Scientific) with 10 % FBS (HyClone), 2 mmol/L GlutaMAX (Gibco), human recombinant interleukin 7 (5 ng/mL, Pepro Tech Inc) and human recombinant interleukin 15 (10 ng/mL, Pepro Tech Inc). Cells were cultured in an incubator (Thermo Fisher Scientific) at 37 °C under an atmosphere of 5% CO_2_ and 90% relative humidity.

**Synthesis of 4-arm-PLGA-Acry**

The LA (7.5 g), GA (2.5 g), pentaerythritol (0.14 g), and tin(II) 2-ethylhexanoate (10.0 mg) were mixed in a 25 mL flask bottle and heated to 130 ºC in oil bath with stirring for 8 h. The product was dissolved with DMF and dialyzed (Cut WM 3,000 Da) for two days in DMF. The 4-arm-PLGA was further precipitated in water as white solid. The 4-arm-PLGA (5.0 g) was dissolved in dichloromethane mixed with TEA (1.0 g), and placed into an ice bath. The methacryloyl chloride (1.0 g) was dissolved in dichloromethane and added to the PLGA solution by drops. Eight hours later, the product was purified by dialysis in DMF for another two days. The final product was obtained by precipitation in water as a white solid. ^1^H NMR (400 M Hz, CDCl_3_, ppm): δ 6.20 (s, 4H), 5.63 (s, 4H), 5.02-5.32 (m, 93H), 4.56-4.94 (m, 74H), 4.17 (s, 8H), 1.95 (s, 12H), 1.35-1.75 (m, 300H). ^1^H NMR indicated the molecular weight of the 4-arm-PLGA-Acry as 10,000 Da, and the efficiency of the methacryloyl chloride modification was close to 100 %.

**Mechanical strength test**

The mechanical strength of PMNs (before etching, after etching, and after loading with CAR T cell) was determined by pressing a stainless-steel plate against the PMNs on an Instron tensile testing machine. The initial gauge was 2.00 mm between the tips of MN and the plate, with 10.00 N as the load cell capacity. The plate approaching MNs speed was set as 0.1 mm/s.

**CAR constructs and generation of CAR-T cells.**

The CSPG4-specifc CAR was constructed by cloning the scFv 763.74, CD8α stalk and transmembrane domain, the 28 intracellular domain, and CD3ζ intracellular signaling domains into the SFG vector. The B7-H3-specific CAR was constructed by cloning the scFv 376.96, CD8α stalk and transmembrane domain, the 28 intracellular domain, and CD3ζ intracellular signaling domains into the SFG vector. The retroviral supernatant was prepared using 293T cells transfected with the plasmid mixture of the retroviral vector, the Peg-Pam-e plasmid encoding MoMLV gag-pol, and the RDF plasmid encoding the RD114 envelop. Supernatant containing the retrovirus was collected 48 and 72 hours after transfection and filtered with 0.45 μm filters. Buffy coats from healthy donors were purchased from the Gulf Coast Regional Blood Center, Houston, TX. Peripheral blood mononuclear cells (PBMCs) isolated with Lymphoprep density separation (Fresenius Kabi Norge) were activated on plates coated with 1 μg/mL CD3 (Miltenyi Biotec) and 1 μg/mL CD28 (BD Biosciences) agonistic monoclonal antibodies (mAbs). On day 2, T lymphocytes were transduced with retroviral supernatant using retronectin-coated plates (Takara Bio). Three days later, T cells were collected and expanded in complete medium (45 % RPMI-1640 and 45 % Click’s medium (Irvine Scientific), 10 % Hyclone FBS (HyClone), 2 mM GlutaMAX (Gibco), 100 unit/mL of Penicillin (Gibco) and 100 μg/mL of streptomycin (Gibco) with IL-7 (10 ng/mL; PeproTech) and IL-15 (5 ng/mL; PeproTech), changing medium every 2-3 days. On day 12-14, cells were collected for *in vitro* and *in vivo* experiments.

**Loading CAR T cells into the porous microneedle patch**

CAR T cells were harvested from the medium and concentrated at 1×10^7^ cells/mL, and the microneedle patch was placed into the CAR T cells suspension under vacuum (100 mbar) for 20 min. Then the microneedle patch was removed and the residual number of CAR T cells was counted by flow cytometry with counting beads (Invitrogen) as an internal standard to determine the loading efficiency. For the observation of CAR T cells loaded into the microneedle patch, CAR T cells were incubated with CFSE (Thermo Fisher, cat. # C34554) for 10 min, then washed twice with PBS. The microneedle patch was labeled with Rhodamine B by adding the dye when preparing the microneedle patch. After loading CAR T into the microneedle patch as introduced above, the microneedle patch was placed in a glass-bottom dish and fixed with a fluorescent mounting medium (Thermo Fisher, cat. # TA-030-FM). The micro distribution of CAR T cells on the microneedle patch was detected by confocal laser scanning microscopy (CLSM, LS880, ZESSI) and reconstructed into 3D graphs with Imaris software.

**Distribution of CAR T cells and WM115 cells in 3D Matrigel model *in vitro*.**

The CFSE labeled WM115 cells (1×10^6^ cells/mL) were dispersed in 1 mL phenol red-free DMEM medium (containing 10% FBS) and mixed with 1 mL phenol red-free Matrigel (Corning) by pipetting. The mixture was transferred to a glass-bottom 96-well plate (50 μL/well) and incubated at 37 °C to form a gel within 24 hours. DIO labeled CAR T cells were administrated to the gels by dropwise, injection, and microneedle delivery, followed by incubation for three days. The distribution of CAR T cells and WM 115 cells were observed with CLSM (LS880, ZESSI).

**Function and viability of the CAR T cells *in vitro* after loaded with microneedle patch**

Luciferase expressing WM115 cells (WM115-Luc, 1×10^5^ cells/well) were incubated with CAR T cells or microneedle loaded CAR T cells (MN@CAR T, 1×10^5^ cells/well), respectively in an ultralow adherent 24-well plate with complete medium. Three days later, the viability of WM115 cells, which is an indicator of the function of the CAR T cells, was evaluated by IVIS® Spectrum *in vivo* imaging system (PerkinElmer) in the presence of luciferase substrate. In addition, the supernatant of the co-incubation medium was collected by centrifugation (1000 g, 10 min, 4 ºC) to determine the cytokine secretion level of the CAR T cells. Human IL-2 and IFN-*γ* secreted by CAR T cells were measured by enzyme-linked immunosorbent assay (ELISA, IL-2, Invitrogen, cat. # 88-7025-22; IFN-γ, cat. # 88-7316-22) according to the manufacturer’s instructions.

For the *in vitro* CAR T cells proliferation evaluation, CAR T cells (1×10^6^) were incubated in the dark with CFSE (final concentration of 1 μM) for 10 min at room temperature. Excess CFSE was washed three times with complete medium before loading the labeled CAR T cells into the microneedle introduced previously. WM115 cells (1×10^5^ cells/well) and CAR T cells or PMN loaded CAR T cells (1×10^5^ cells/well) were co-cultured in an ultralow adherent 24-well plate for three days with complete medium. The T cells were stained with PE-CD3 (Biolegend, cat. no. 300308, clone HIT3a), and CAR T cells proliferation was detected by flow cytometry (BD LSRFortessa).

**Xenograft WM115 melanoma and pancreatic tumor generation.**

The NOD.Cg-Prkdc^scid^ Il2rg^tm1Wjl^/SzJ (NSG) mice (female, 6-8 weeks) were purchased from the Jackson laboratory. All animal experiments were performed in compliance with an animal study protocol approved by the Institutional Animal Care and Use Committee at University of California, Los Angeles. WM115 (5×10^6^) cells were injected into the NSG mice subcutaneously. The xenograft melanoma tumor grows to the size of 100 mm^3^ within one-month after the inoculation of the WM115 cell. Before a treatment or other tumor-associated experiments, the solid tumor was resected, leaving ~10% tumor to mimic residual micro tumor after surgery.

For orthotopic pancreatic tumor model generation, 2×10^5^ FFLuc-Panc01 cells were suspended in 20 μL PBS, mixed with 20 μL Matrigel (Corning) and injected into the pancreas of the NSG mice after surgical exposure of pancreas. The Panc01 tumor cells were carefully inoculated into the tail of the pancreas and the wound on the abdomen was carefully closed in two layers, with running 4-0 Vicryl, and polypropylene sutures for the skin.

**CAR T cells application *via* microneedle *in vivo***

The CAR T cells loaded-microneedle patch was clamped with a tweezer and the tips of the microneedle were applied toward the solid tumor. In the melanoma tumor model, the microneedle was directly pressed at the tumor site for insertion. In the pancreatic tumor model, a soft damper was employed to assist microneedle insertion.

**Distribution of CAR T cells in tumor.**

CAR T cells were labeled with DIO according to the manufacturer’s instructions, then the CAR T cells were applied to the WM115 melanoma tumor by intratumor injection or microneedle delivery. Twenty-four hours later, the tumors were harvested and embedded in OCT to obtain frozen sections. The slides of the tumor section were observed with CLSM (LS880, ZESSI) to display the distribution of CAR T cells in the tumor.

**CAR T cell proliferation *in vivo*.**

CAR T cells (1×10^6^) with luciferase expression were administrated to the residual tumor by subcutaneous injection, intratumoral injection or PMN injection. After application, the bioluminescence signals were recorded on day 0, day 3, day 6, day 9 and day 12 with IVIS (Perkin). Signal analysis was performed by Living Image Software. Twelve days after application, the tumors were harvested, digested into single cells, and labeled with anti-human CD3 antibody (Biolegend, cat. no. 300308, clone HIT3a) to count the number of CAR T cells by flow cytometry. The cytokines secretion by CAR T cells, including human IL-2 and human IFN-γ, were analyzed with ELISA (Invitrogen) according to the manufacturer’s instructions.

**Immunofluorescence staining**

Tumors were harvested from the mice and froze in the optimal cutting temperature (OCT) medium before cutting *via* a cryotome. 20 μg/mL Alexa Fluor® 594 anti-human CD8a antibody (Biolegend, cat no. 100758) and Alexa Fluor® 488 anti-human CD4 antibody (Biolegend, cat no. 317408) was used to staining the tumor slices overnight at 4 °C. The tumor cell apoptosis was analyzed with terminal deoxynucleotidyl transferase dUTP nick end labeling (TUNEL) assay kit following the standard protocol.


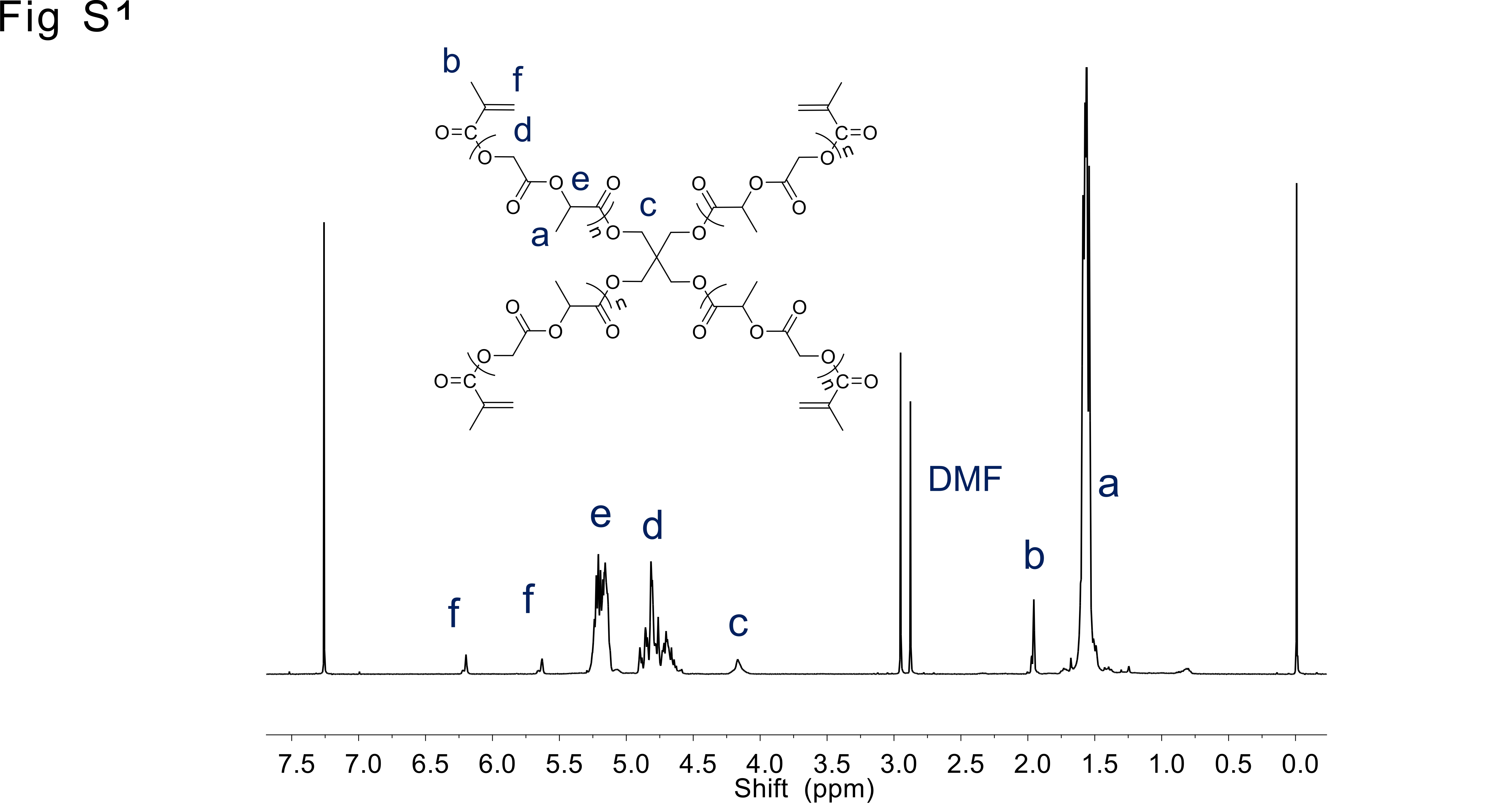


Figure S1. The ^1^H NMR spectra of the 4-arm-PLGA.


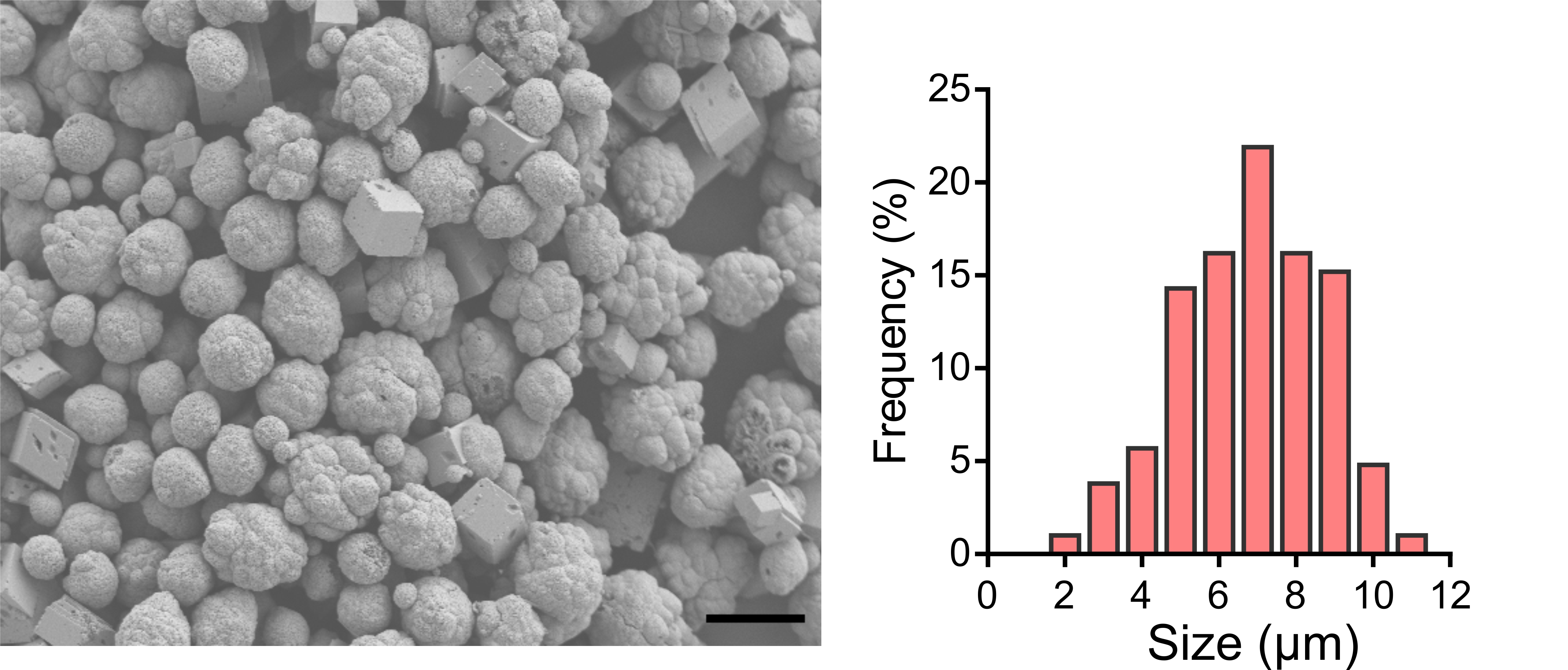


Figure S2. The SEM image and size distribution of the CaCO_3_ microparticles, scale bar: 10 μm.


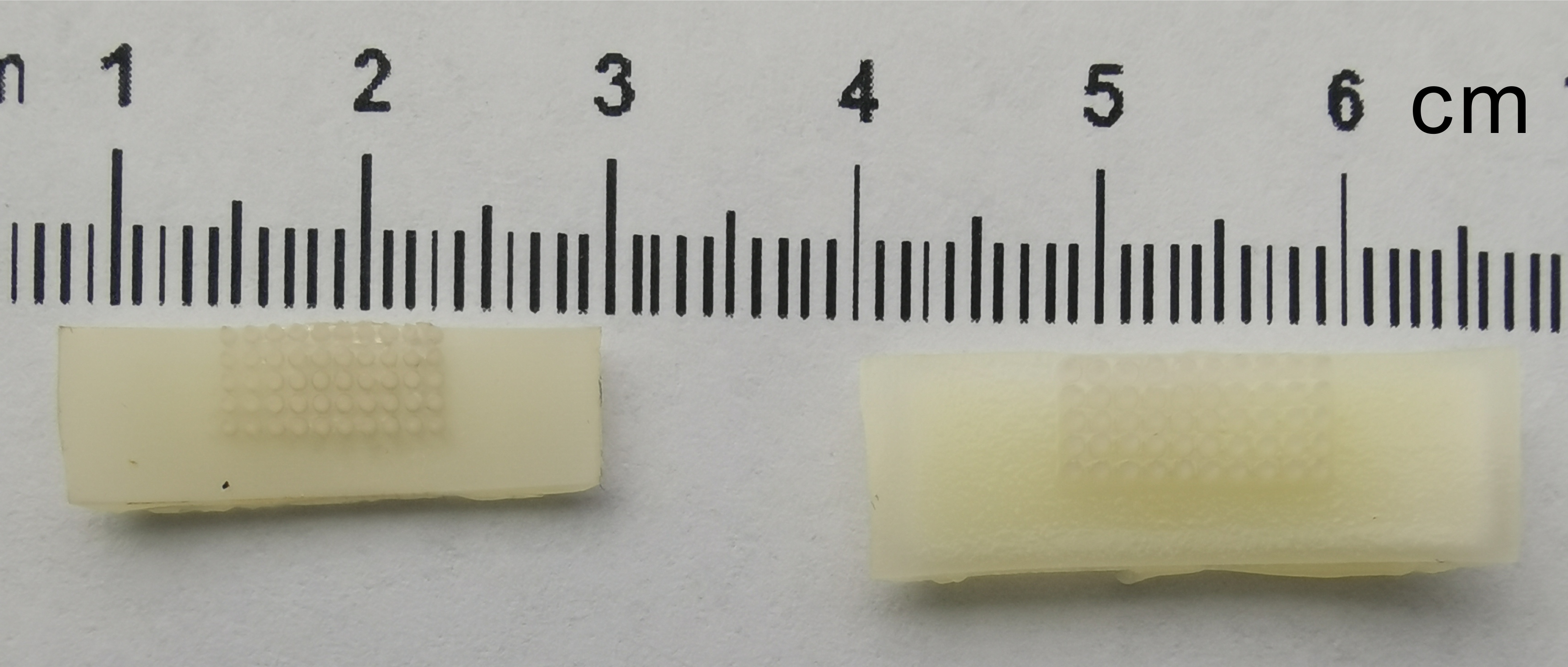


Figure S3. The image of CaCO_3_ containing microneedle patch before (left) and after (right) swelling in 1,4-dioxane.


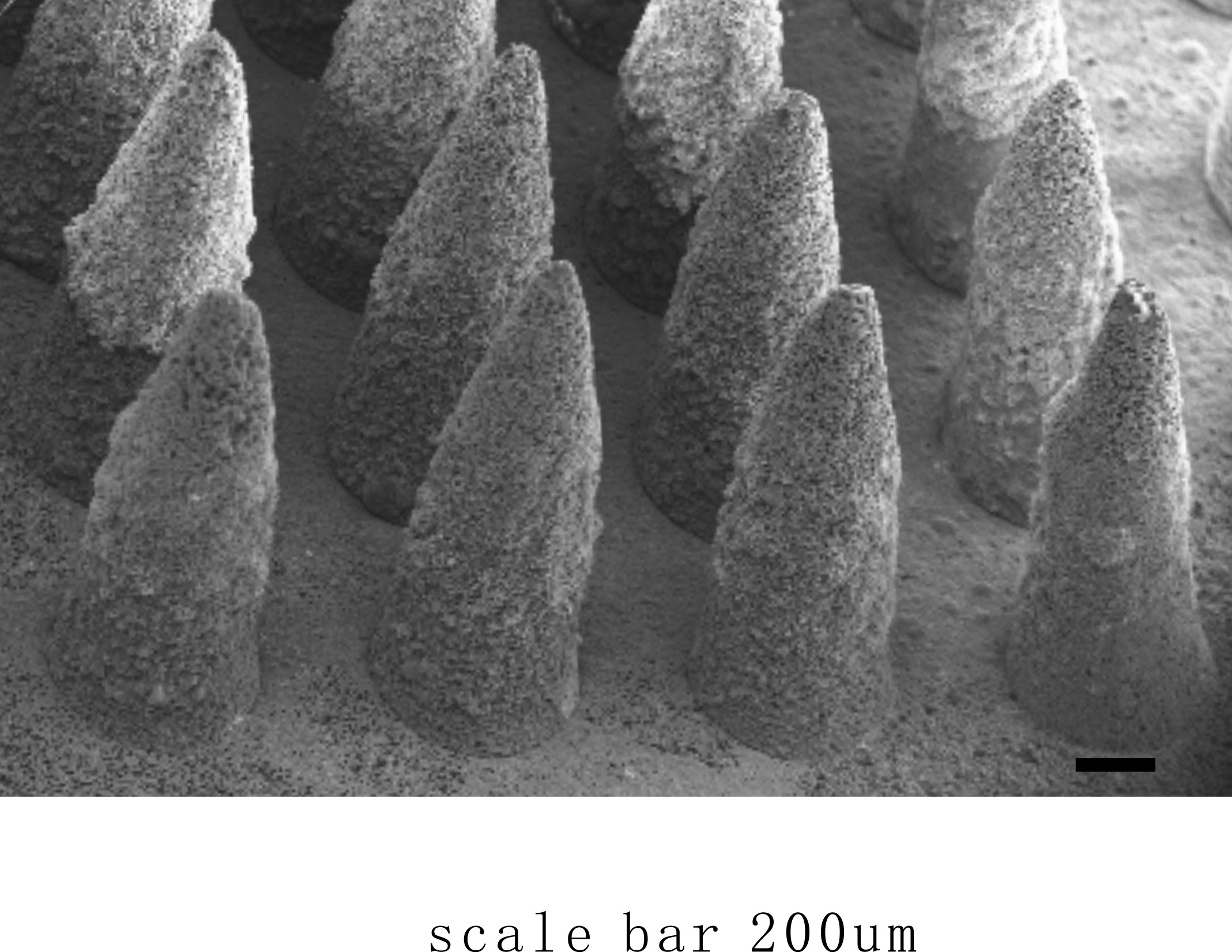


Figure S4. SEM image of PMN, scale bar: 200 µm.


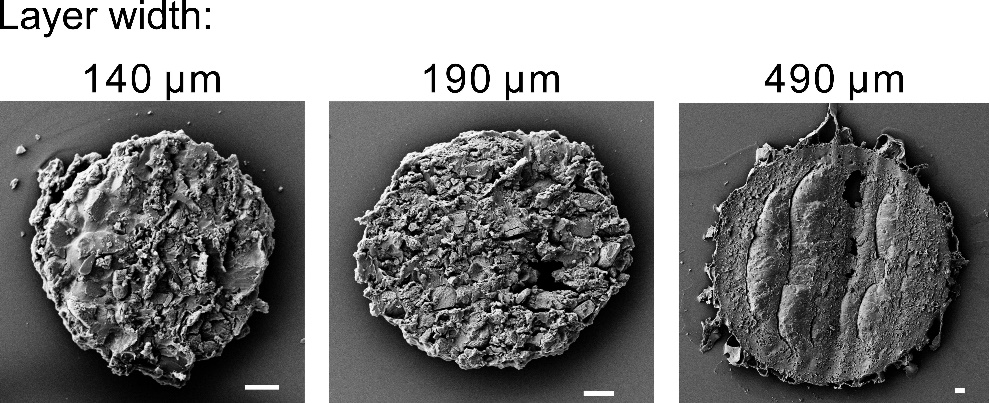


Figure S5. SEM images showing the cross-sections of the unetched PMN, scale bar: 20 µm.


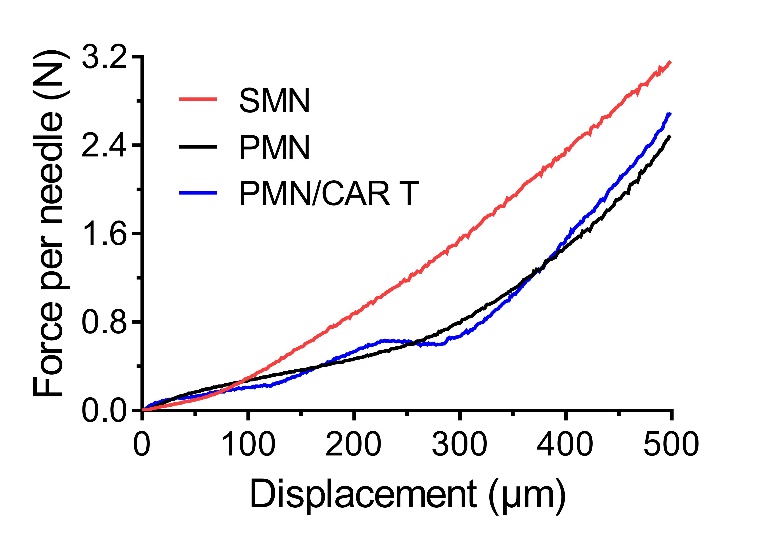


Figure S6. Mechanical strength of unetched solid microneedle (SMN), porous microneedle (PMN) and CAR T cells loaded PMN (PMN@CAR T).


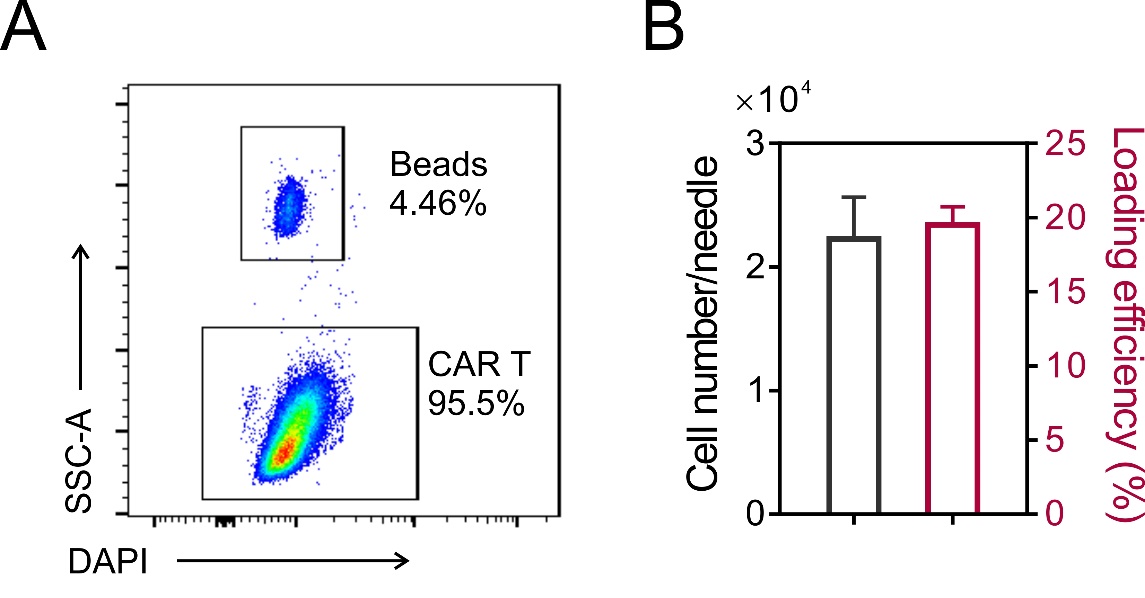


Figure S7. CAR T cells loading in PMN. (A) Representative flow cytometry quantification of CAR T cells calculated with counting beads. (B) The loading amount and loading efficiency of CAR T cells in the PMN


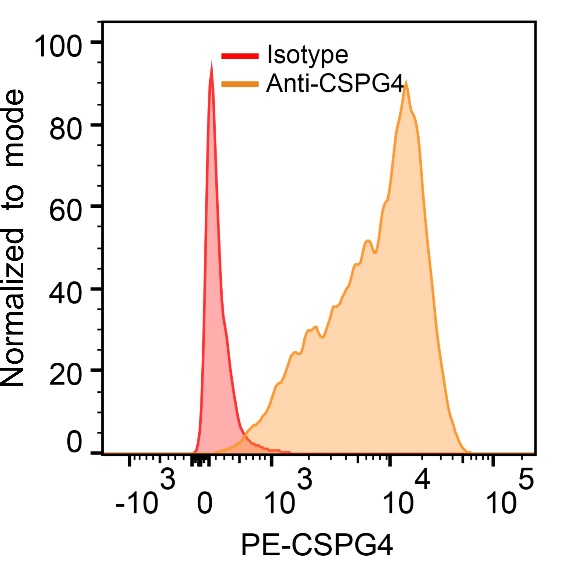


Figure S8. Flow cytometry analysis of CSPG4 expressed on WM115 cells.


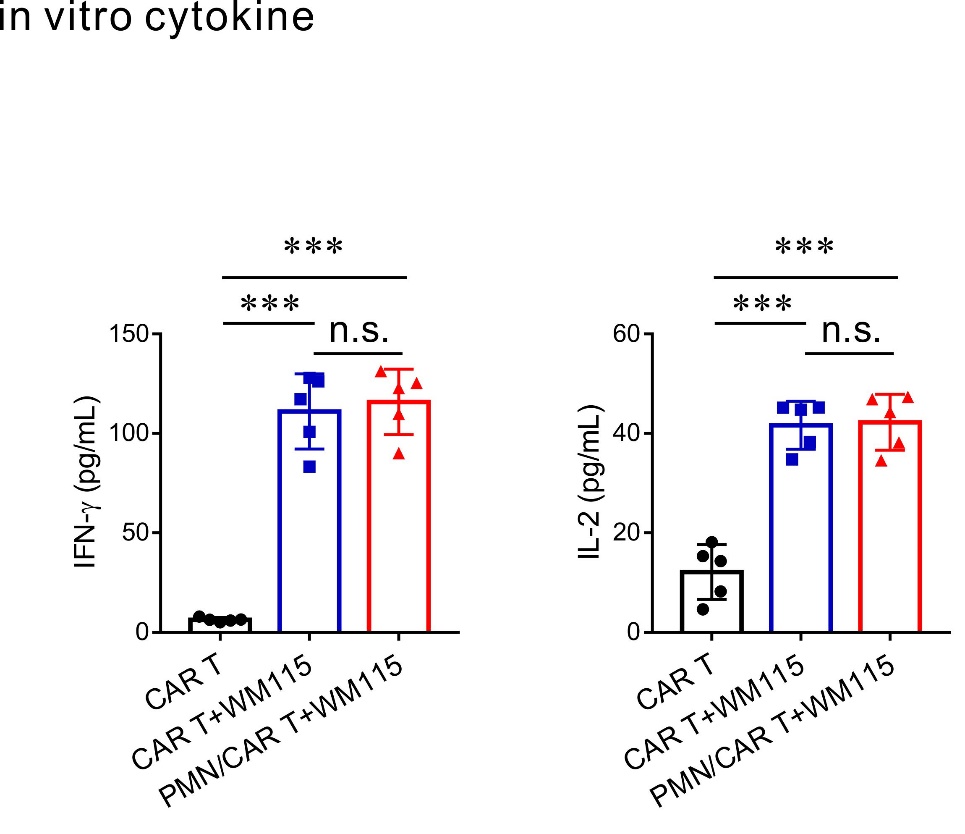


Figure S9. Cytokine level of human IFN-*γ* and IL-2 after T cells co-incubation with WM115 cells in different conditions, n = 5. Data are presented as mean ± s.d., statistical significance was calculated *via* one-way ANOVA with a Tukey post-hoc test. *P value*: ****P* < 0.001, n.s. means no significant difference.


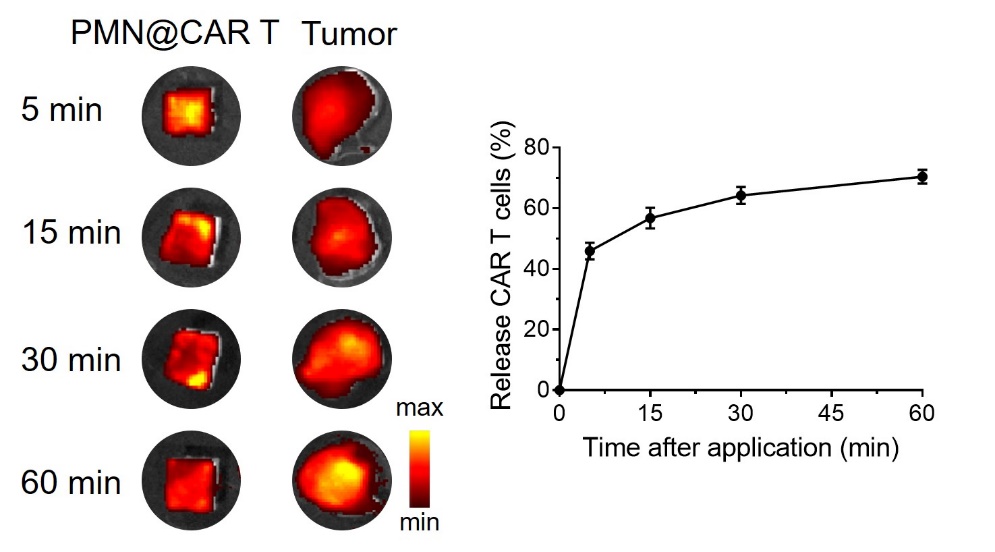


Figure S10. CAR T cells release from PMN after application into WM115 tumor *in vivo*.


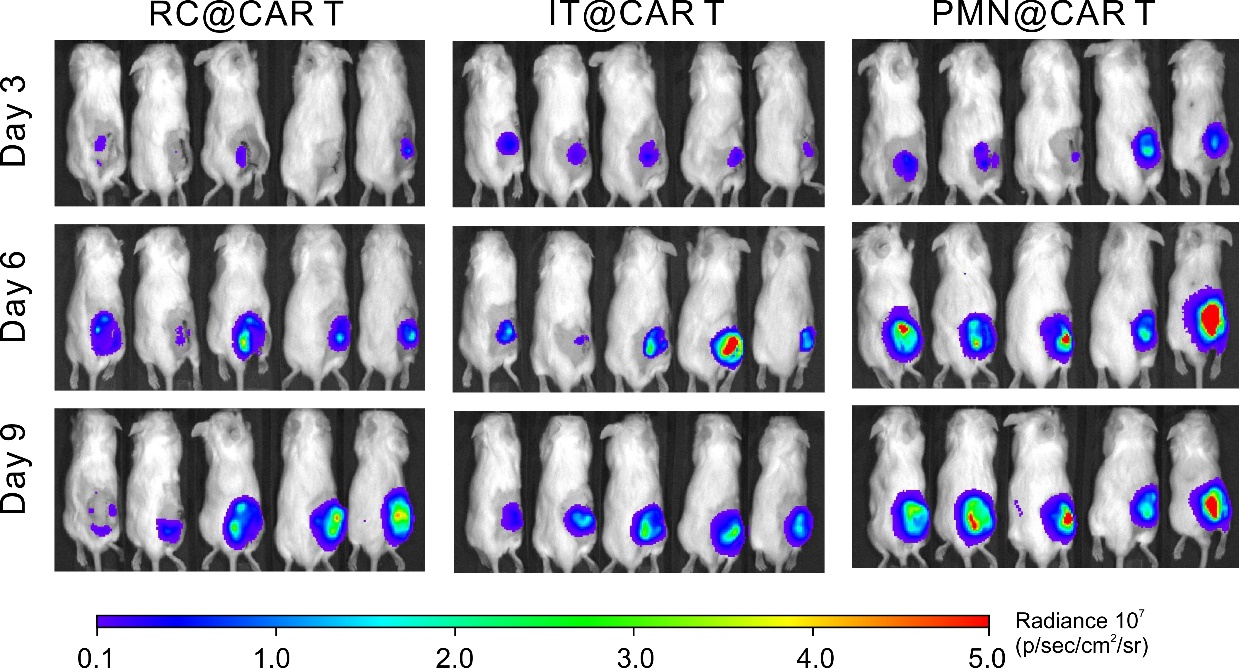


Figure S11. CAR T cells proliferation *in vivo*. Bioluminescence images of mice administered with luciferase-expressing CAR T cells at the tumor site on day 3, day 6, and day 9 post-surgery.


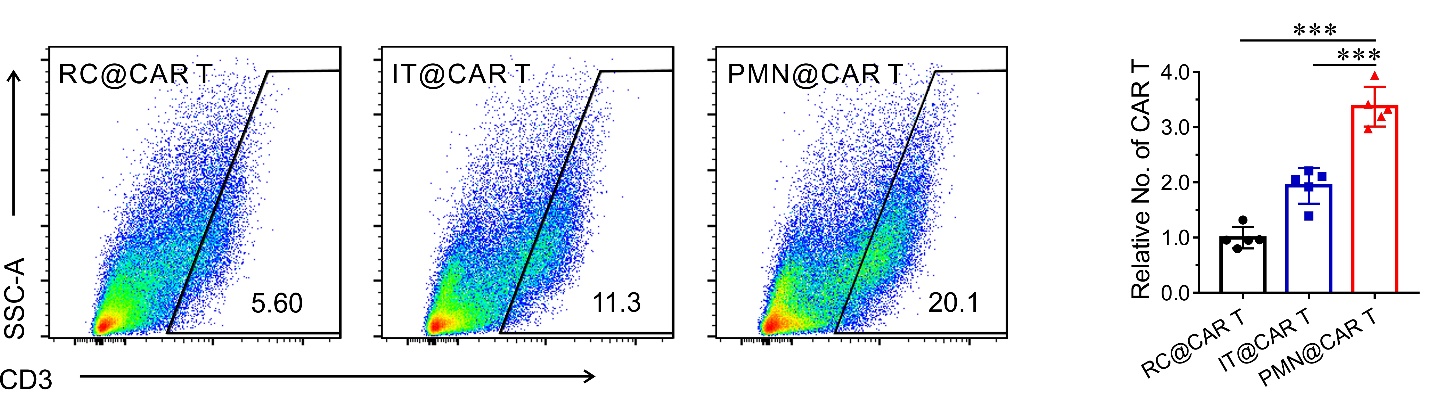


Figure S12. Representative flow cytometry of CD3 positive T cells in tumor 12 days after treatment with CAR T cells, n = 5. Data are presented as mean ± s.d., statistical significance was calculated *via* one-way ANOVA with a Tukey post-hoc test. *P* value: ****P* < 0.001*.*


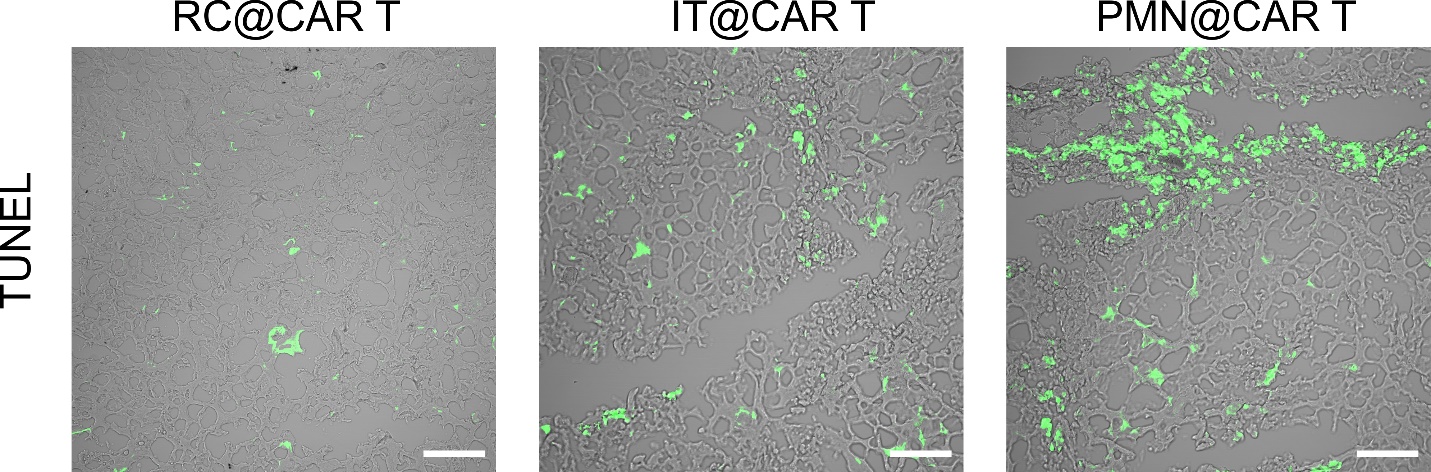


Figure S13. Representative immunofluorescence images of tumors showing apoptosis cells after treatment with TUNEL, scale bar: 100 μm.

**Movie S1 (separate file)**. A representative video is showing the etching of CaCO_3_ microparticles from the microneedle in HCl/hexane solution.

**Movie S2 (separate file)**. A representative video is showing the *in vitro* distribution of CAR T cells (red) in the 3D tumor model mimicking hydrogel by dropwise administration.

**Movie S3 (separate file)**. A representative video is showing the *in vitro* distribution of CAR T cells (red) in the 3D tumor model mimicking hydrogel by intra-gel injection.

**Movie S4 (separate file)**. A representative video is showing the *in vitro* distribution of CAR T cells (red) in the 3D tumor model mimicking hydrogel by PMN-assistant administration.
